# Analyzing Retinal Vessel Morphology in MS Using Interpretable AI on Deep Learning-Segmented IR-SLO Images

**DOI:** 10.3390/bioengineering12080847

**Published:** 2025-08-06

**Authors:** Asieh Soltanipour, Roya Arian, Ali Aghababaei, Fereshteh Ashtari, Yukun Zhou, Pearse A. Keane, Raheleh Kafieh

**Affiliations:** 1 Medical Image and Signal Processing Research Center, Isfahan University of Medical Sciences, Isfahan 817467346, Iran; asieh.soltanipour1365@gmail.com (A.S.); aaghababaei78@gmail.com (A.A.); 2 Department of Engineering, Durham University, South Road, Durham DH1 3LE, UK; royaarian101@gmail.com; 3 School of Medicine, Isfahan University of Medical Sciences, Isfahan 7346181746, Iran; 4 Isfahan Neurosciences Research Center, Isfahan University of Medical Sciences, Isfahan 7346181746, Iran; f_ashtari@med.mui.ac.ir; 5 NIHR Biomedical Research Centre for Ophthalmology, Moorfields Eye Hospital NHS Foundation Trust and UCL Institute of Ophthalmology, London EC1V 2PD, UK; yukun.zhou.19@ucl.ac.uk (Y.Z.); p.keane@ucl.ac.uk (P.A.K.); 6 Centre for Medical Image Computing, University College London, London WC1E 6BT, UK; 7 Department of Medical Physics and Biomedical Engineering, University College London, London WC1E 6BT, UK

**Keywords:** multiple sclerosis, scanning laser ophthalmoscopy, segmentation, deep learning, feature extraction, machine learning, feature importance

## Abstract

Multiple sclerosis (MS), a chronic disease of the central nervous system, is known to cause structural and vascular changes in the retina. Although optical coherence tomography (OCT) and fundus photography can detect retinal thinning and circulatory abnormalities, these findings are not specific to MS. This study explores the potential of Infrared Scanning-Laser-Ophthalmoscopy (IR-SLO) imaging to uncover vascular morphological features that may serve as MS-specific biomarkers. Using an age-matched, subject-wise stratified k-fold cross-validation approach, a deep learning model originally designed for color fundus images was adapted to segment optic disc, optic cup, and retinal vessels in IR-SLO images, achieving Dice coefficients of 91%, 94.5%, and 97%, respectively. This process included tailored pre- and post-processing steps to optimize segmentation accuracy. Subsequently, clinically relevant features were extracted. Statistical analyses followed by SHapley Additive exPlanations (SHAP) identified vessel fractal dimension, vessel density in zones B and C (circular regions extending 0.5–1 and 0.5–2 optic disc diameters from the optic disc margin, respectively), along with vessel intensity and width, as key differentiators between MS patients and healthy controls. These findings suggest that IR-SLO can non-invasively detect retinal vascular biomarkers that may serve as additional or alternative diagnostic markers for MS diagnosis, complementing current invasive procedures.

## 1. Introduction

Multiple sclerosis (MS) is an immune-mediated disease of the central nervous system (CNS) characterized by chronic inflammation, demyelination, gliosis, and axonal degeneration [1]. MS most frequently affects young adults, with about 2.3 million people suffering from the disease worldwide [1]. It presents with a variety of signs and symptoms, including limb weakness and paresthesia, autonomic nervous system dysfunction, and visual impairment [2]. To date, numerous retinal changes, both structural and functional, have been reported in MS eyes, even in the absence of any history of optic neuritis [3]. A large number of optical coherence tomography (OCT) studies have revealed that the retinal nerve fiber layer (RNFL) and ganglion cell-inner plexiform layer (GCIPL) become thinner in MS patients, compared to healthy control (HC) individuals [4]; this has also been observed in other neurodegenerative conditions like Alzheimer’s disease (AD) [5] and Parkinson’s disease (PD) [6]. Indeed, the retina, as a unique window to study brain pathology, may contain potential markers for diagnosing MS, without the need to employ current invasive, costly, and time-consuming diagnostic procedures like magnetic resonance imaging and lumbar puncture [2].

Infrared reflectance scanning laser ophthalmoscopy (IR-SLO) is an imaging technology often performed along with OCT to lock B-scans at a fixed position, thus enhancing image quality by mitigating the impact of eye motion-induced noise during image acquisition. Additionally, IR-SLO technology enables ophthalmologists to observe disease progression and response to treatments at longitudinal follow-up visits [7]. IR-SLO works by illuminating the retina with a laser beam in a raster pattern, and creating en-face two-dimensional images using the backscattered light passed through a confocal aperture [8]. The resulting images look very similar to conventional fundus photographs which is why IR-SLO is also known as monochromatic fundus photography. However, IR-SLO images pose different contrast characteristics, meaning that some structures that are not obvious in fundus photographs may show up well using IR-SLO images; conversely, there could be imperceptible regions in IR-SLO images where fundus photography captures well. This is because each imaging modality uses varied wavelengths [8].

To date, artificial intelligence (AI) has been extensively employed in the diagnosis of MS [9,10,11,12,13], relying on brain imaging data, clinical findings, laboratory variables, and OCT thickness measurements Ongoing research during recent years have shown that besides the conventional finding of inner retinal thinning, other retinal pathologies, such as vascular changes, may also occur in MS, serving as possible candidates for automated, non-invasive diagnosis of the disease. Statistical studies based on retinal imaging modalities that include vascular information, such as fundus camera photography and OCT angiography, have revealed reduction in retinal vessel diameter [14] or decreased vessel density (VD) in the superior capillary plexus (SCP) within parafoveal and peripapillary regions [15,16,17,18].

Recently, IR-SLO images have also been shown to effectively discriminate between MS and HC states. For example, Bolton et al. demonstrated that asymmetry between the right and left eyes in IR-SLO images can be used to distinguish MS patients from HCs [19]. Similarly, Aghababaei et al. reported a classification accuracy of 88% when discriminating between 164 MS and 150 HC IR-SLO images [20]. In a more recent study, Arian et al. showed that incorporating IR-SLO images alongside OCT thickness maps significantly improves diagnostic performance, achieving an accuracy of 96.85 ± 0.45, sensitivity of 100 ± 0.0, and specificity of 94.96 ± 0.66—compared to an accuracy of 94.32 ± 1.12, sensitivity of 97.59 ± 0.43, and specificity of 90.48 ± 1.86 when using OCT alone [9]. These results suggest that the high diagnostic capability of IR-SLO–based models may be attributed, at least in part, to vascular biomarkers present in these images; however, this remains a hypothesis and warrants further investigation.

Despite these promising results, the interpretability of AI models applied to IR-SLO and other fundus-like images has not been adequately addressed. In today’s increasingly AI-driven medical landscape, the ability to trust, understand, and explain AI decisions is crucial. Although convolutional neural networks (CNNs) often achieve high diagnostic accuracy, they typically operate as black boxes, offering limited insight into the features driving their predictions [21]. To ensure clinical acceptance, especially among neurology and ophthalmology professionals, it is therefore essential to identify and present the specific discriminative features within IR-SLO images—particularly those related to the optic disc and retinal vasculature—in a transparent and clinically interpretable manner.

In machine learning, analyzing feature importance is a valuable task that assesses the contribution of each feature to the model’s decision-making process, thereby enhancing the interpretability of AI-based medical diagnoses [22]. FI measurements may also serve as a key preprocessing step to select the most relevant features so that the risk of model overfitting to high-dimensional input data becomes minimized [23]. In the field of ophthalmology, various FI methods have been employed to identify the most important parameters from OCT, OCT-A, fundus photography, fundus autofluorescence, and indocyanine green angiography data, for classifying diabetic retinopathy [24,25,26], diabetic macular edema, choroidal neovascularization, drusen [27,28], glaucoma [29,30], retinopathy of prematurity [31], retinoblastoma [32] and also cognitive impairment [33,34]. Regarding MS, FI methods like SHapley Additive exPlanations (SHAP) [35], recursive feature elimination (RFE) [36] and decision tree-based methods have been utilized in two recent works that were aimed to discriminate MS from HC [37,38] and other CNS demyelinating diseases [38] based on OCT thickness measurements.

**Figure 1:**
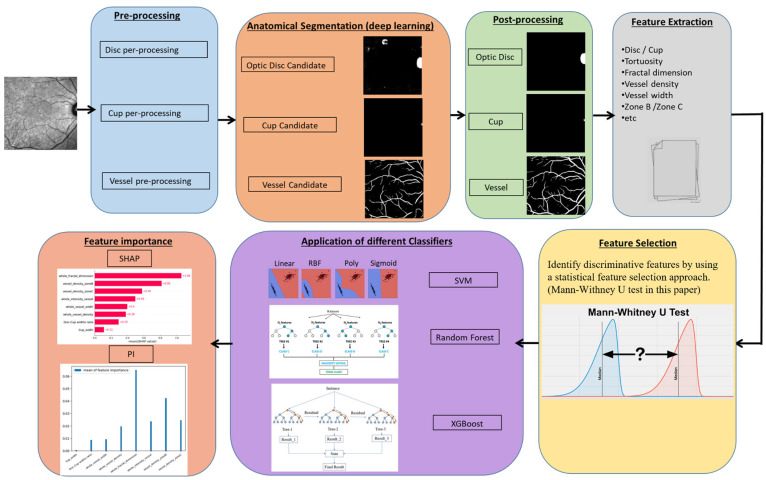
Overview of the proposed method to analyze the morphological changes in SLO images related to MS.

In the current study, our aim is to investigate whether IR-SLO images exhibit distinctive features specific to MS, enabling differentiation between MS patients and healthy controls (HC). To this end, we first performed automated segmentation of the optic disc, optic cup, and retinal vessels by adapting a deep learning model originally trained on color fundus images to the IR-SLO modality. This adaptation highlights the potential of leveraging existing fundus-trained models for IR-SLO applications, particularly when IR-SLO-specific data are limited—an important advantage in data-constrained research settings. Based on these segmentations, multiple image processing algorithms were applied to extract several features concerning the optic disc (cup width, disc width, and cup-to-disc ratio) and retinal vessels (e.g., VD, fractal dimension [FD], vessel intensity (VI) and vessel width (VW)). Following this, a statistical feature selection (FS) process was employed to identify the most discriminative features. These selected features were then utilized as inputs to three ML classifiers: random forest (RF), extreme gradient boosting (XGBoost), and support vector machine (SVM)—solely to facilitate feature importance analysis. Importantly, the goal of incorporating these classifiers was not to develop ML-based diagnostic models but rather to evaluate and rank the contribution of each feature using a popular feature importance (FI) method. This study represents the first comprehensive analysis of IR-SLO images focused on identifying the most clinically relevant vessel morphological features for differentiating MS from HC. A brief overview of our proposed approach is illustrated in Figure 1.

Key contributions of this study include:

- Adaptation and validation of a deep learning model trained on fundus images for accurate segmentation of optic disc, cup, and retinal vessels in IR-SLO, demonstrating effective cross-domain transfer when IR-SLO-specific data are limited.
- Comprehensive feature importance analysis using SHAP across multiple machine learning models to identify key markers differentiating MS from healthy controls.
- The first detailed morphological assessment of IR-SLO images in MS, highlighting potential non-invasive retinal biomarkers for diagnosis.

## 2. Materials and Methods

### 2.1. Dataset

#### 2.1.1. Internal Dataset

In this study, we utilized the Isfahan dataset as the internal dataset, which comprised of OCT scans and IR-SLO images from MS patients and HC individuals. This dataset was captured using the Heidelberg SPECTRALIS SD-OCT device (Heidelberg Engineering, Heidelberg, Germany). The Isfahan dataset, acquired from a prior study by Ashtari et al. [39], included a total of 280 IR-SLO images. Among these, 146 images were from 71 patients with MS, and 134 images were from 35 HC individuals. The study took place between April 2017 and March 2019 at the Kashani Comprehensive MS Center in Isfahan, Iran, which serves as a primary referral center for MS in the region.

#### 2.1.2. External Dataset

To evaluate the generalization capability of our proposed model, we also used the publicly available Johns Hopkins dataset as an external dataset from an independent center. This dataset, acquired using the same SD-OCT device (Heidelberg Engineering, Heidelberg, Germany), included IR-SLO and OCT images from the right eyes of 35 individuals, consisting of 14 HCs and 21 MS patients [40].

#### 2.1.3. Test, Validation, and Train Data Splitting

To prevent any leakage among test, validation, and training samples, a subject-wise approach was adopted, wherein all images associated with a specific subject were exclusively designated for either the test, validation, or training set [41].

To create the internal test dataset, stochastic matching based on age and gender was employed so that the potential confounding effects of these variables are minimized. This was done before proceeding with the subsequent separation of training and validation data [42]. Initially, 20% of subjects with MS were randomly chosen and designated as the test dataset. For each selected MS case, an HC patient with the closest age and the same gender was also included in the age-gender matching test dataset.

Following this, the splitting of train and validation data was conducted using k-fold cross-validation (CV) on the remaining patients [9]. K-fold CV is preferred over a random split for its completeness and generalization and ensures that the entire dataset is utilized for training. In this method, predictive models are evaluated by dividing the dataset into k folds and training and evaluating the model k times, each time using a different fold as the validation set. In this study, the number of folds (k) was set as 5. Moreover, to maintain an equal proportion of certain labels (MS or HC) in each fold, stratified sampling was employed.

#### 2.1.4. Data Augmentation

Data augmentation is a well-known technique in ML studies used to artificially expand the size of a limited training dataset to mitigate the risk of over-fitting. This involves making minor alterations to the existing training dataset to create new and plausible examples. In this study, several geometric and color space transformations were performed, including vertical and horizontal flips, height and width shifts within the range of ±5 pixels, rotation within the range of ±15 degrees, and adjustments to image brightness in the range of 0.8 to 1.5.

### 2.2. Feature Extraction

In this study, basic ML models were trained with manually extracted features related to the optic disc, cup, and vessel morphology, rather than utilizing deep learning models. This approach provided a clearer insight into the clinical significance of different features discriminating between MS and HC. To accomplish this, the initial steps involved the segmentation of the optic disc, optic cup, and vessels from the IR-SLO images, as discussed in Section 2.2.1 and Section 2.2.2.

#### 2.2.1. Anatomical Segmentation

Considering the limited size of our IR-SLO image dataset and the absence of corresponding ground truth, we leveraged the pre-trained anatomical segmentation models outlined in [43]. These models had been trained on public datasets comprising fundus photographs with associated ground truth. However, there are inherent differences between IR-SLO and fundus photography modalities as the former uses a single-wavelength laser light but the latter employs red, green, and blue wave bands) [44]; therefore, the models trained with fundus photographs [43] may not perform well on our dataset consisting of IR-SLO images utilized as the test set. Consequently, we implemented several pre-processing and post-processing techniques to tailor the segmentation for the optic disc, cup, and vessels in IR-SLO images, explained in the following sections.

To achieve optic disc localization and segmentation, we employed the pre-trained LW-Net model outlined in [43]. However, as mentioned earlier, adjustments were made Optic Disc Segmentation to the model outputs to make them applicable to the monochromatic IR-SLO images. The optic disc segmentation phase utilized in this study comprises three primary sub-stages: pre-processing, identification of the optic disc candidates, and post-processing. Detailed explanations of each stage are provided below.

##### Pre-Processing

Typically, the optic disc is recognizable as a bright yellowish or white area in color fundus images, appearing brighter than the background when the image is converted to grayscale. Conversely, in IR-SLO images it takes on a different appearance, presenting as a dark region that is darker than the background. To address this difference, in the initial stage, the pixel intensity values in IR-SLO images were inverted, swapping black pixels with white and vice versa. As a result of this adjustment, the optic disc manifested as bright regions in the IR-SLO images.

In this study, many IR-SLO images exhibited variations in brightness and uneven illuminations, potentially leading to inaccuracies in identifying optic disc candidates in subsequent stages. To tackle this problem, we employed a specific IR-SLO image as a reference, characterized by a discernible optic disc and minimal intensity fluctuations. The IR-SLO image selected as a reference is displayed in the Supplementary Figure S1. Subsequently, in order to manipulate the intensity distributions and mitigate contrast level variations in other IR-SLO images, their histograms were matched with the histogram of the chosen reference image.

In IR-SLO images, the central reflection of blood vessels is observable in both arteries and veins [44]. This reflection tends to be more pronounced in arteries, especially when dealing with color fundus images. In certain inverted IR-SLO images obtained from the previous step, the central reflections in veins, i.e., thicker and more transparent around the optic disc compared to the arteries, were observed as dark strips with an intensity level nearly comparable to that of the cup. This phenomenon poses the risk of generating inaccurate optic disc candidates in subsequent stages. To mitigate this effect, two morphological closing and opening operations with a rectangular structure element were applied to the intensity of the inversed IR-SLO images obtained from the previous stage. The IR-SLO image that represents these central reflections as dark strips in veins, along with the image resulting from the removal of their effects is depicted in the Supplementary Figure S2.

Finally, every IR-SLO image was resized to (512,512) to accommodate the large batch size required by the LW-Net model. The application of the LW-Net model in identifying optic disc candidates is explained in detail in the following section [43].

##### Optic Disc Candidates

To identify candidate regions for the optic disc in the IR-SLO images following the pre-processing stage, we utilized the LW-Net model, which comprises two U-Nets as described in [43]. However, as mentioned earlier, the monochromatic nature of IR-SLO images, in contrast to the 3 color channels of fundus images used in [43] as a dataset, necessitated adjustments and modifications in the output of the second U-Net within LW-Net. Initially, the IR-SLO images were utilized as inputs for the encoder, corresponding to the first U-Net in the LW-Net. The outputs of the decoder, associated with the second U-Net in the LW-Net, were classified into three categories, namely, background, optic disc, and cup. Specifically, the second channel resulting from the decoder was considered as the segmentation map for optic disc candidates. Finally, we examined these candidates to delineate the optic disc area.

##### Post Processing

During this stage, the binary images containing the optic disc candidates were evaluated based on distinct characteristics such as shape, bounding box, and coordinates. The segmentation of the optic disc was finally completed by undertaking the following five main sub-stages:

- In order to eliminate noise pixels situated between the candidates, particularly in low-quality IR-SLO images exhibiting intensity variations despite applying the histogram matching method, morphological closing and opening operations, using a structural element in the shape of an ellipse, were performed on the binary images containing the candidates.
- Since the optic disc appears as bright areas in the inverted images that are generated during the pre-processing phase, dark regions cannot be identified as the optic disc. To filter out candidate areas with a low probability of being the optic disc, candidates with a mean intensity lower than a specific threshold were excluded. This threshold was determined based on the mean intensity values of all candidates in each image.
- Candidate regions in each image with an area smaller than a certain threshold were excluded (2300 pixels for candidates located on either side of the images and 3000 pixels for those located near the center of the images).
- The shape and area of the remained candidates were determined using connected component analysis, and those with a line shape or a low width-to-length ratio in their bounding box were eliminated. Candidate regions close to the center of the images were further filtered by removing those with a low length-to-width ratio within their bounding box. Ultimately, the final optic disc candidate was identified as the one with the greatest area.
- The final optic disc candidate underwent a blob detection algorithm to delineate the boundary of the optic disc. To achieve this, an ellipse transform was employed, considering that in some images only one arc of the optic disc may be visible. The algorithm used to calculate the boundary and width of the optic disc candidate positioned on the sides of the IR-SLO images is summarized in the Supplementary Figures S3 and S4.

##### Cup Segmentation

To localize and segment the cup in the IR-SLO images, we utilized the outcome obtained from optic disc segmentation stage. Initially, a window surrounding the segmented optic disc in the original data (not the inversed version) was selected and then used as input for the first U-Net in the LW-Net model. The first channel of the LW-Net model output was considered a segmentation map for cup candidates. Subsequently, the mean intensity value of each candidate was compared to a certain threshold, i.e., the mean intensity of all candidates in each image. If the intensity value of a candidate was smaller than the threshold, it was eliminated. Following this, within each image, the center of the bounding box for each remaining candidate and its distance from the center of the segmented optic disc were computed. The cup candidate with the smallest distance was chosen as the final candidate, provided it adhered to three primary criteria, unless it needed to be excluded:

1. The bounding box of the candidate should entirely fall within the optic disc boundary.
2. The width-to-length or length-to-width ratio of the candidate must be less than 2, as the cup does not have a narrow oval shape.
3. The area of the candidate must exceed a specific threshold, set at 700 in this work.

Ultimately, should a cup candidate be present, the boundary of the cup would be established using an ellipse transform, akin to the method outlined in the optic disc segmentation section.

##### Vessel Segmentation

To segment the vessels in IR-SLO images, we employed the pre-trained method proposed in [43], complemented by a post-processing step crucial for accurately segmenting blood vessels. A detailed explanation of these two main stages is provided below.

##### Binary Vessel Segmentation Map

Because of uneven illumination and consequent intensity variations among the IR-SLO images in the dataset we used, our initial step involved employing the histogram matching algorithm using a proper reference image. This reference image, displayed in the Supplementary Figure S5, is characterized by minimal intensity changes, served to eliminate variations in brightness and prevent the segmentation of false candidate pixels that may represent vessels.

Afterward, we employed the SEGAN model designed in [43], which is a variant of U-Net comprising a segmentor and a discriminator trained using an adversarial learning strategy. The IR-SLO images, having undergone histogram matching, were initially resized to (912, 912) to alleviate the computational demands before being used as inputs for the SEGAN model. The output from the discriminator provided a segmentation map, where each pixel represented the likelihood of being a blood vessel. In this study, pixels with a likelihood greater than 0.3 were chosen to create the binary representation of blood vessels.

##### Post Processing

There were some discontinuities in the segmented blood vessels in our dataset resulting from the implementation of the SEGAN model [43]. To address this issue, we employed a post-processing step and leveraged two useful algorithms: a region-growing method and a missing algorithm [45], to fill in the discontinuous parts of the segmented blood vessels. In the first stage, to overcome the discontinuities, especially along the major blood vessels, we implemented the region-growing algorithm, which is briefly explained in the Supplementary Figure S6.

To implement the region-growing algorithm, for each terminal point with an intensity greater than a specified value (40 in this study), we examined the intensity of its neighboring pixels not belonging to segmented vessels. Those pixels whose brightness difference with a specified threshold was less than a predetermined value (10 in this work) selected as candidate points. The candidate point with the lowest brightness difference value was then chosen as the new seed. It is worth noting that the threshold value for selecting candidate points was the intensity of the terminal point, and this value became updated by averaging the intensity of both the terminal point and the newly selected seed.

For each selected new seed, the aforementioned algorithm was repeated until no neighboring point satisfied the criterion. The Supplementary Algorithm S1 details our proposed region growing approach, where “p_i_” represents the ith terminal point, “S_1_” is a set of neighboring points satisfying a certain criterion explained above, and “P_S1,i_” represents ith point in “S_1_”.

In the subsequent step, we applied the missing algorithm proposed in [45] to address any remaining small discontinuities in the segmented vessels from the initial stage by utilizing the vessel graph and identifying landmark points on it.

#### 2.2.2. Feature Measurement

In this study, a series of clinically relevant manual features were calculated from the segmented optic disc, optic cup and blood vessels to classify individuals into two MS and HC classes. Optic disc width, optic cup width and the ratio of disc width to cup width were the feature sets extracted from the optic disc and cup. In addition, the features including average width, FD [46], VD, VI, VW, distance measure tortuosity [47], squared curvature tortuosity [47], tortuosity density [48], and linear regression tortuosity were calculated from the extracted blood vessels in the whole image, zone B and zone C. Zone B and zone C represent the area located between 0.5 to 1 and 0.5 to 2 optic disc diameter (OOD) from the disc margin, respectively, as described in [49]. A visual depiction of these zones and the clinically manual feature used in this study are shown in the Supplementary Figure S7 and Table S1.

### 2.3. Feature Selection

FS is a critical preprocessing step in ML aimed at identifying and retaining relevant features while removing non-informative or redundant ones. This enhances the model’s predictive accuracy, robustness, and generalizability by focusing on significant predictors, thereby reducing the risk of overfitting [50,51].

FS methodologies are broadly classified into three categories: filter methods, wrapper methods, and embedded methods. Filter methods assess feature relevance based on their statistical correlation with the target variable, independent of any ML algorithms. They utilize statistical measures to evaluate the relationship between each input variable and the target variable, selecting those with strong correlations for further analysis.

Wrapper methods use a specific ML model to evaluate different subsets of features. They iteratively score and select feature subsets based on their predictive performance, often employing a greedy approach to find the best combination of features. Embedded methods integrate FS directly into the model training process, performing FS simultaneously with model training, often through regularization techniques. Models that inherently support FS, such as decision trees and regularized regression models, particularly benefit from embedded methods [52].

In this study, we applied a statistical-based filter method to our dataset for FS, divided into groups diagnosed with MS and HC. We computed the mean values of each feature across all IR-SLO images for individual subjects. Given the non-normal distribution of clinical features, we employed the Mann-Whitney U test to identify significant features differentiating these groups.

The Mann-Whitney U test is a robust non-parametric statistical technique used to compare two independent samples. It evaluates whether the distributions of two groups differ significantly by comparing their ranks. A key element of the Mann-Whitney U test is the *p*-value. If the *p*-value is less than the significance level (commonly set at 0.05), it suggests that the difference between the groups is statistically significant, and the null hypothesis can be rejected [53].

### 2.4. Feature Importance

FI methods assign a score to each feature within a feature set, indicating the feature contribution to the final model performance. This not only offers a comprehensive insight into the behavior of the model but also facilitates the ranking and comparison among features. Generally, ML interpretation can be divided into two main categories: global and local explanations. Global explanation aims to decode the model as a whole and explain the behavior of the model across the entire data distribution, while the goal of local explanation is to interpret the predictions of the model for individual observations [54].

In this study, we employed SHAP [55] a well-known FI technique that provides both global and local explanations for model decisions, based on the cooperative game theory. In this approach, to measure the impact of feature  i, two models are trained: $f_{S\cup \left\{i\right\}}(x_{S\cup \left\{i\right\}})$ is the first trained model with feature i and $f_{S}(x_{S})$ is the second trained model without feature i. Then the Shapley value for feature i, denoted by $\emptyset_{i}$, is mathematically defined as the following formula:

(1) $$\emptyset_{i}\left(x\right)=\;\sum_{S\subset F⧵\{i\}}\frac{\left|S\right|!\left(\;\left|F\right|-\;\left|S\right|-1\right)!}{\left|F\right|!}\;\left[f_{S\cup i}\left(x_{S\cup i}\right)-\;f_{S}(x_{S})\;\right]$$

where, x = the observation input, $\emptyset_{i}(x)$ = Shapley value for the feature i, the observation x, and the model f, F = a coalition of features that does not include feature I, $\left|F\right|$ = the size of the coalition, $f_{S}$ = the trained model on the subset of features S, $f_{S\cup i}$ = the trained model on the subset of features S and i, $x_{S}\;$ = the restricted observation of x given the subset of features S, and $x_{S\cup i}\;$ = the restricted observation of x given the subset of features S  and i. Hence, a Shapley value can be defined as the average marginal contribution of a feature within all possible combinations of features. In addition to providing both local and global explanations, SHAP stands out as a consistent method for measuring FI, as SHapley values remain unchanged even when the model undergoes alterations unless the contribution of a feature changes. Therefore, SHAP has gained popularity as one of the most promising and dependable tools for FI measurement.

Since the SHAP method requires a preliminary classification process, we applied three conventional ML models—SVM [55,56], Random Forest (RF) [57], and XGBoost [58]—using the statistically selected features (Section 2.3). The Optuna optimization library was used to tune their hyperparameters [56]. The best-performing model (XGBoost) was then used as the basis for SHAP-based feature importance analysis.

The optimized hyperparameters for all three classifiers are summarized in Supplementary Figure S8 and Table S2.

#### Evaluation of the Classifiers

To assess the performance of the above classifiers, we employed a set of metrics, including accuracy (ACC), sensitivity (SE), specificity (SP), precision (PR), and F1-score (harmonic mean between precision and recall), using the following mathematical formula:

(2) $$ACC=\frac{TP+TN}{TP+TN+FP+FN}$$

(3) $$SE=\frac{TP}{TP+FN}$$

(4) $$SP=\frac{TN}{TN+FP}$$

(5) $$PR=\frac{TP}{TP+FP}$$

(6) $$F_{1}=\frac{2\times TP}{2\times TP+FP+FN}$$

where TP, FN, TN, and FP represent the metrics for true positive, false negative, true negative, and false positives, respectively. Furthermore, receiver operating characteristics (ROC) were employed to depict the relationship between true and false positive samples, while precision-recall curves were utilized to illustrate the trade-off between PR and recall.

### 2.5. Comparison of Demographic Characteristics

To compare the demographic characteristics of individuals from the two datasets, independent Student’s *t*-test (or its non-parametric equivalent in case the variable distribution is not normal according to the Kolmogorov-Smirnov test) and chi-square test were utilized for age and gender, respectively. *p*-values less than 0.05 were considered significant.

All the experiments in this study were conducted using Python programming language in a Python 3.7 software environment; for deep learning applications, the backend Torch platform was utilized.

All the experiments in this study were implemented using Python programming language, in the Keras platform backend in python 3.7 software environment (Code and models are available at: https://github.com/Asieh65/Interpretable-AI-in-IR-SLO-images (accessed on 31 July 2025).

## 3. Results

In this study, 280 IR-SLO images (146 MS, 134 HC) from 106 subject groups (35 MS, 71 HC) were utilized as an internal dataset (the Isfahan dataset), and 35 IR-SLO image (21 MS, 14 HC) from 35 subjects were used as an external dataset (the Johns Hopkins dataset). The demographic characteristics of individuals from the Isfahan (35 MS, 71 HC) and Johns Hopkins (21 MS, 14 HC) centers are depicted in Table 1.

To construct a test dataset based on the age-gender matching algorithm explained in Section 2.1.1, 10 MS patients and 10 HC individuals with the same gender and closest age were selected. The remaining images were then utilized for splitting data into the training and validation sets using stratified k-fold CV (k = 5).

### 3.1. Segmentation

As mentioned in Section 2.2.1 and Section Optic Disc Candidates, we modified the LW-Net model proposed in [43] to ensure accurate segmentation of IR-SLO images in our dataset, involving both pre- and post-processing stages. Table 2 illustrates the quantitative results of the segmentations. The ground truth for each segmented structure was provided by an expert ophthalmologist.

**Figure 2:**
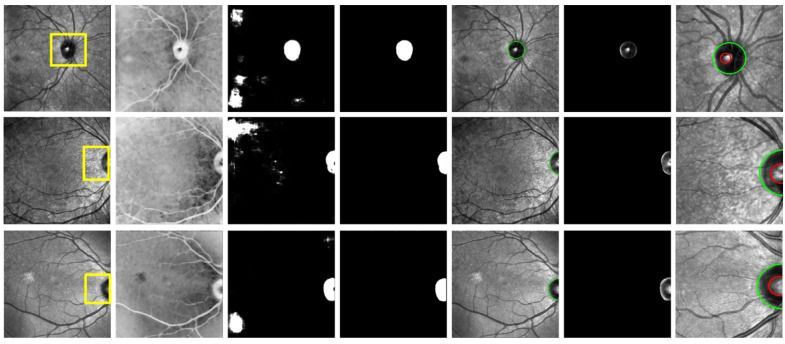
Visualization results for optic disc and cup segmentation. The columns from left to right display original sample IR-SLO images, pre-processing step for disc segmentation, optic disc candidates, post-processing step for disc segmentation, windows surrounding the segmented optic disc, and finally, the optic disc and cup segmented by green and red circles, respectively. Note that the last column shows the zoomed state of the yellow window on the original SLO images in the first column.

Visualization results for optic disc and cup segmentations are depicted in Figure 2. The optic disc was successfully segmented irrespective of whether it was located on the left or right side of the image, with only an arc of its circular border visible.

**Figure 3:**
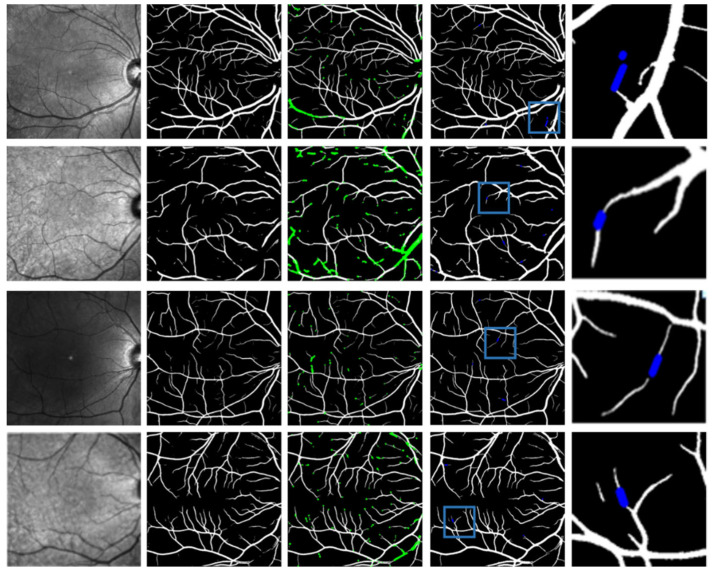
Visualization of the vessel segmentation results for four samples, where the columns from first to last indicate the original SLO images, the segmentation algorithm outcomes, the region growing outcomes, the missing algorithm results, and the zoomed-in results of the missing stage for a small window of the images, respectively.

The segmentation outcomes for blood vessels in the IR-SLO images are also illustrated in Figure 3. The results of the region-growing and missing algorithms are showcased in the third and fourth columns, respectively. The fifth column depicts the zoomed-in results of the missing stage for a small window of the images.

### 3.2. Feature Selection

Using a significance level set at *p* < 0.05, this statistical analysis identified eight discriminating features between MS and HC individuals, namely optic cup width, disc-cup width ratio, VW, VD, FD, VI, VD in zone B, and VD in zone C.

### 3.3. Feature Importance

As discussed in Section 2.4, the selected features were given to three ML classifiers, i.e., SVM, RF, and XGBoost (Section 3.3.1), and the model with optimal performance was utilized for calculating SHAP values (3.3.2).

#### 3.3.1. Classification

The classification performance of SVM, RF, and XGBoost using features from IR-SLO images of MS and HC individuals is shown in Table 3. The corresponding hyperparameters optimized using the Optuna are displayed in the Supplementary Table S2. It is evident that the XGBoost is the winning method (ACC = 82.37%; area under the ROC [AUROC] = 86.28%; area under the precision-recall curve [AUPRC] = 85.37%; F1 = 82.14%; SP = 78.63%; SE = 83.79%, precision recall (PR) = 80.39%). Therefore, we selected XGBoost as the foundational classifier to quantify FI in the subsequent stages of our analysis.

#### 3.3.2. SHAP Calculations

**Figure 4:**
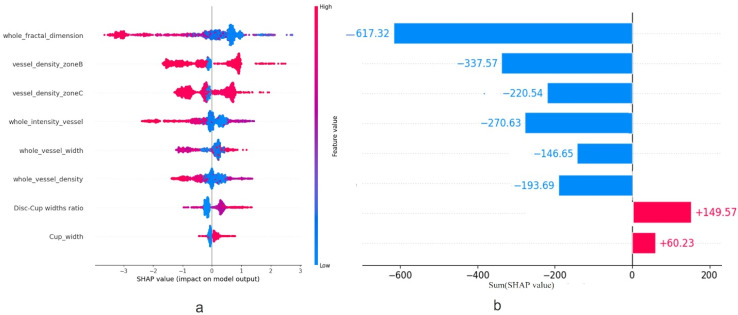
Overview of the SHAP values for the designed XGBoost model; (**a**) illustrates the contribution of the features for each observation, while (**b**) shows how each feature impacts the model output, positively or negatively.

**Figure 5:**
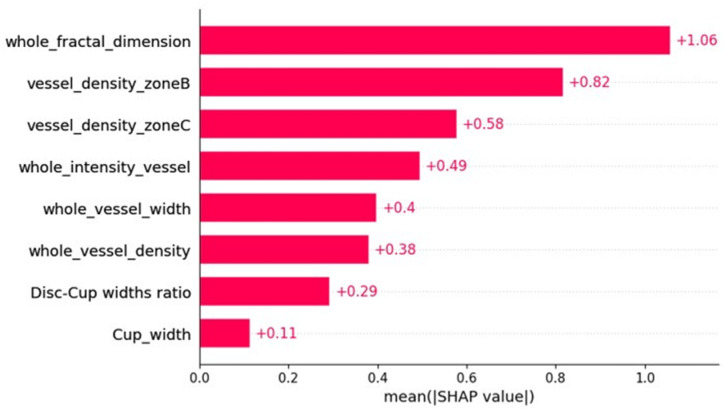
Overview of the important features measured by the SHAP method.

As mentioned earlier in Section 2.4, SHAP is a consistent and objective way to measure the impact of each feature, offering a global and local insight into the output of the ML model. Figure 4a,b represents a summary plot of the importance of each feature using SHAP, when XGBoost, i.e., the best model for MS and HC classification, is employed. In this figure, the x-axis and y-axis represent the SHapley values and the features utilized, respectively. Additionally, each dot demonstrates the SHapley value of a specific feature for a given observation. The color of the dots demonstrates the value of the feature, ranging from low (blue color) to high (red color). Features with positive SHapley values contribute positively to the prediction, guiding the model towards predicting MS, whereas those with negative SHAP values exert a negative influence, leading the model to predict HC. The magnitude of these values represents the strength of their effect on the model outcomes. Additionally, different feature values can be linked to both positive and negative Shapley values; for instance, the majority of red points in whole FD, whole VI, whole VD and whole VW rows have a negative Shapley value, suggesting that a higher value of these features is associated with a reduced probability of being categorized as MS. In Figure 5, the features are ordered based on their importance, with the x-axis representing the average of the absolute SHAP values for each feature across all observations.

### 3.4. Generalization

**Figure 6:**
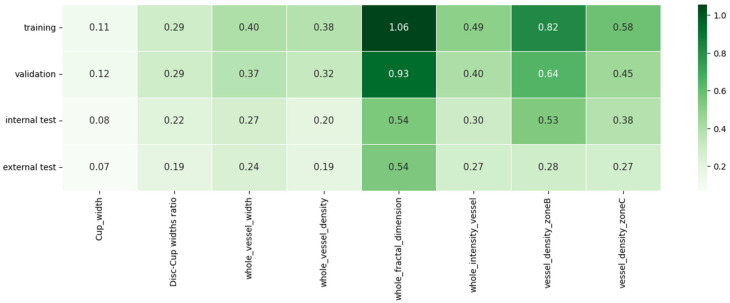
Visualization of FI on training, validation, internal test, and external test datasets using SHAP approach.

To assess the generalization capability of our model, we examined the FI scores generated using the SHAP approach across training, validation, internal test, and external test datasets. As illustrated in Figure 6, the top five features identified were in the same order across each of the four different datasets. This high level of consistency in FI measurements highlights our model’s ability to generalize effectively, ensuring that it can deliver accurate and reliable predictions, regardless of data variations.

### 3.5. Robustness Analysis

**Figure 7:**
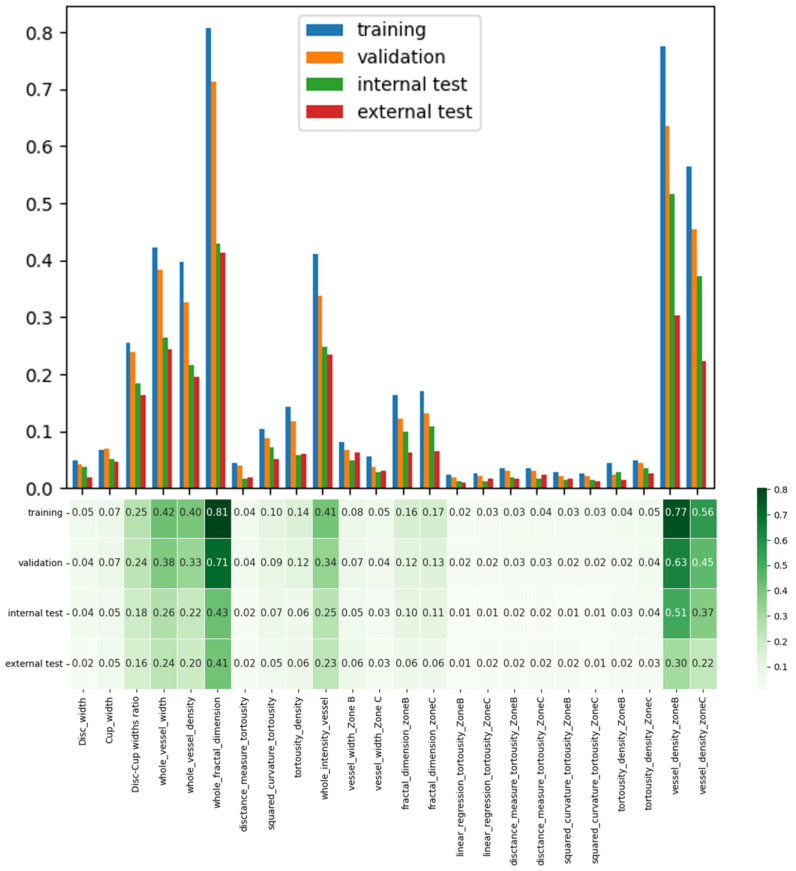
Visualization of important features obtained from the entire set of clinical features across training, validation, internal test, and external test datasets, in the absence of the feature selection stage. The identified important features are approximately in the same order as the important feature set obtained in Section 3.4.

To evaluate the robustness of the FI methods, we also computed the FI scores for the entire set of features derived in Section 2.2.2, without applying Mann-Whiteny U test prior to FI measurement across training, validation, internal test, and external test datasets. We observed that the five discriminative features obtained from the entire clinical feature set were nearly identical to those identified after employing statistical FS, approximately in the same order of importance. Figure 7 illustrates the FI-score for five important features, considering both the entire set of clinical features and those obtained by the Mann-Whiteny U test. This similarity underscores the steadfast reliability and credibility of our model’s FI assessment even without getting help from statistical models.

In the subsequent phase, we assessed the performance of the XGBoost model by employing varying numbers of features, based on their importance. We illustrated that utilizing the first five important features as input for the model yielded the highest accuracy (ACC = 81%) compared to other combinations. This satisfactory model performance is also similar to that achieved when the informative feature set (number of features = 8) is employed.

**Figure 8:**
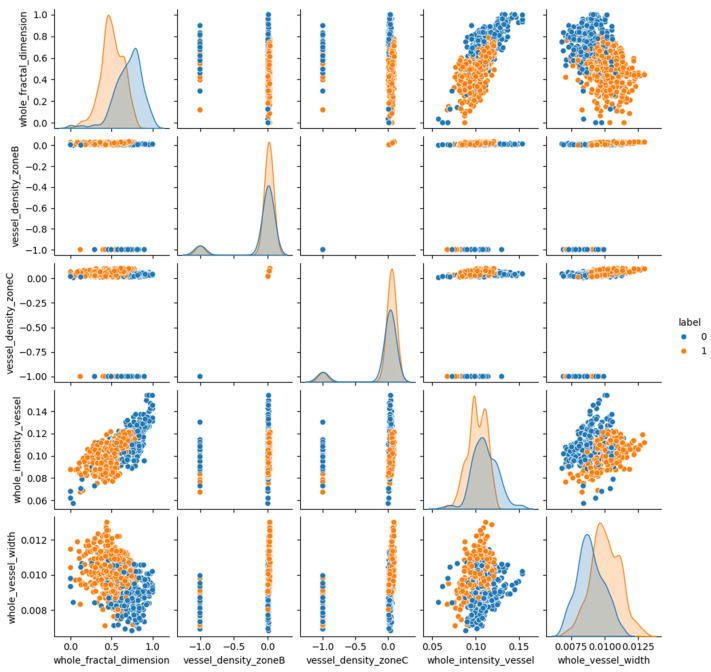
The distribution and relationship between the first five important features obtained by SHAP method. Each point represents a sample: red dots correspond to MS patients (label = 1), and blue dots correspond to normal controls (label = 0).

**Figure 9:**
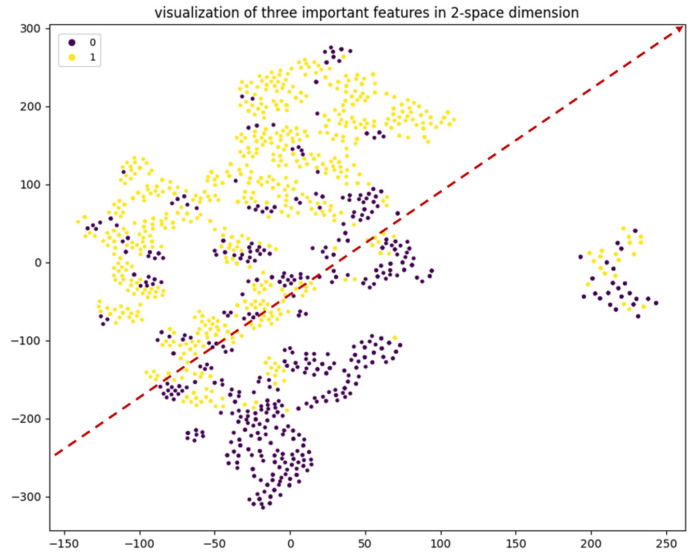
Visualization of five important features in 2-space dimension using t-SNE. Label 0: HC images, label 1: MS images.

So, it was observed that the initial five most important features, namely, total FD, VD in zone B, VD in zone C, total VI and total VW, exhibit the most alterations in IR-SLO images of MS patients, compared to those of HC individuals. The distribution and relationship between these five important features are also shown in Figure 8, with each subplot concisely depicting the relationship between the paired features in the corresponding row and column. Next, we utilized t-distributed stochastic neighbor embedding (t-SNE), known as an unsupervised non-linear dimensionality reduction technique for data exploration (Figure 9), aimed at discovering patterns in lower-dimensional [59].

## 4. Discussion

The strong deep learning-based segmentation performance achieved in this study forms the foundation for the subsequent analysis of retinal biomarkers. We adapted a convolutional neural network originally trained on color fundus photographs to segment optic disc, optic cup, and retinal vessels in IR-SLO images—an imaging modality with significantly different visual characteristics. Despite this domain shift, the model achieved high segmentation accuracy, with quantitative evaluations showing excellent agreement with expert ophthalmologist annotations. These results demonstrate that the adapted deep learning model is highly effective for IR-SLO image analysis and provides precise anatomical delineation essential for extracting clinically meaningful vascular and structural features.

The successful adaptation of a deep learning model trained on color fundus photographs to IR-SLO images is a key advancement of this study. It demonstrates that models developed on large fundus datasets can be effectively repurposed for IR-SLO, even when IR-SLO-specific data are limited. This cross-modality transfer enables high-quality segmentation and opens new opportunities for applying deep learning in underutilized imaging domains. Such adaptability is particularly valuable in clinical research settings where data collection is resource-constrained.

**Figure 10:**
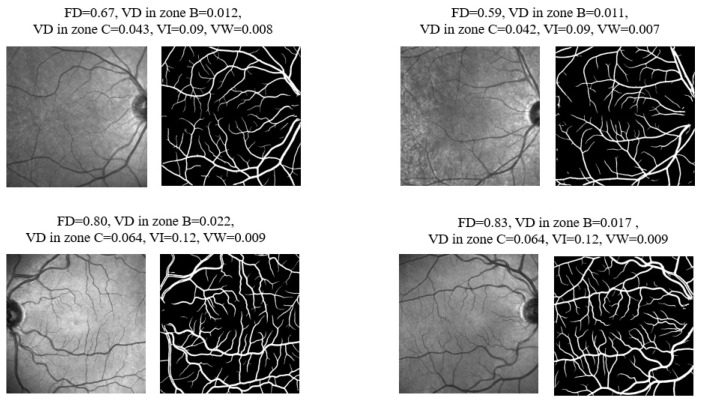
Representation of the first five important features on the images with MS (**first row**) and the HC images (**second row**).

For the first time, we showed that IR-SLO images harbor valuable features that can be used for distinguishing between MS and HC states. Notably, certain retinal characteristics, such as vessel tortuosity and FD, have not been previously investigated in an MS population, even when considering studies on fundus camera photographs. The most important features identified encompass the vascular FD, and VD of Zone B, followed by VD of Zone C, VI and VW of the whole image. Figure 10 provides a visual comparison of the five most important features between MS and HC IR-SLO images, where ten images of the test dataset that were correctly classified by the XGBoost model are illustrated. Evidently, this figure highlights the lower values of FD, VD in zone B, VD in zone C, VI and VW in MS images. The choice of these five features is noteworthy because, when the SHAP method was applied to four different datasets (Section 3.5) and even without initial statistical feature selection (Section 3.5), these five features consistently stood out as the most important.

### 4.1. From Imaging to Insight: Clinical Relevance of Selected Retinal Features

The important features for discriminating MS from HC individuals concerned with retinal vasculature. FD, a measure of branching pattern complexity of vessels, was identified as the second most important parameter, with lower values in MS, similar to cognitive impairment [60,61], stroke [60], hypertension [49], and other systemic/ocular disorders [62]. Furthermore, previous OCT-A studies have revealed that VD of superficial/deep capillary plexuses in macular and/or peripapillary regions becomes reduced in MS patients, especially in those positive for ON, compared to HC individuals [15,16,17]. VI, defined as the ratio between the brightness of vessels to that of the background, showed decreased values in the MS group and served as the fourth highly discriminating parameter. Of note, retinal ganglion cell atrophy due to neurodegenerative processes leads to a decrease in metabolic demand that consequently may result in a reduced blood flow supplying the retina, via autoregulatory mechanisms; this can explain lower values of FD, VD, and VI among MS patients. In this regard, a number of fundus photography studies have shown a decreased retinal blood flow (using a retinal function imager) [63,64] or vessel diameter [65] in MS. In a recent paper, Kallab et al. calculated peripapillary capillary density, large VD, and RNFL/GCIPL thickness in a population consisting of 16 MS patients with a history of unilateral ON and 18 HC individuals [66]. Using a retinal vessel analyzer [67], they extracted central retinal artery and vein equivalents (CRAE and CRVE) and arterio-venous ratio (AVR), with CRAE and AVR shown to be significantly lowered among MS patients, in eyes both with and without antecedent ON [66]. Furthermore, they showed an impaired oxygen metabolism in ON-positive retinas [66]; such finding has also been observed in other retinal oximetry studies [68,69]. Interestingly, altered oxygen consumption may have caused vessels to look less bright and contributed to lower VI values in the MS group. This is because the oxygenated and deoxygenated hemoglobulin molecules have different absorption characteristics of laser light under an IR-SLO source [70]. Finally, vascular changes observed in MS retinas can be considered primary damage, not necessarily secondary to neurodegeneration occurring during the disease course. Studies have shown that the hypoxia consequent to such vascular damage may itself lead to inflammation and demyelination of retinal nerves [71,72]. Hypoxia can lead to inactivation of nuclear factor kappa B, i.e., a key molecule in regulating inflammatory responses; conversely, inflammation can cause endothelial cell damage and vessel occlusion, resulting in hypoxia [71]. Indeed, the hypothesis “hypoxia-inflammation cycle” proposed by Yang and Dunn [71] highlights the fact that these two phenomena have a reciprocal relationship forming a positive feedback loop, where the causal order is unclear [72].

### 4.2. Feature Importance Methods in Retinal Imaging

Similar to our work, FI methods like SHAP [24,37], local interpretable model-agnostic explanations [29,32], RFE [38], and those embedded in tree-based classifiers, e.g., RF [26,29,33,34] and XGBoost [29,33], have been employed in studies on retinal imaging data to identify features specific to various retinal pathologies. Hernandez et al. trained RF, XGBoost, and EBM models with RNFL and GCL thickness values, organized into 64 thickness points and 6 thickness zones. After the classification process, the authors employed the SHAP method, showing features located in zones 1 and 2, corresponding to the temporal regions of GCL, were the most important parameters [37]. Like XGBoost, EBM (https://interpret.ml, accessed on 31 July 2024) is a tree-based algorithm that can analyze FI simultaneously with the classification process. EBM was shown to be more biased when compared to SHAP, as non-clinically relevant areas were also selected by this algorithm [37]. SHAP has also been utilized in other studies on OCT thickness measurements [32] and OCT-A parameters [24] to highlight crucial features in classifying glaucoma and DR, respectively. Additionally, the importance of each pixel within fundus images [32,65] and OCT-B scans [55,66] for detecting retinoblastoma, retinopathy of prematurity, choroidal neovascularization, diabetic macular edema, and drusen has been calculated using SHapley values. In another study on MS patients, RFE was employed as a feature selection technique [38], where Kavaklioglu et al. utilized 24 OCT thickness values (RNFL, GCIPL, and total retina in macular and peri-papillary regions) to classify MS from HC and other CNS demyelinating diseases in a pediatric population. They showed that the thickness of superotemporal GCIPL and temporal RNFL had the greatest contribution to final model predictions [38].

### 4.3. Limitations

This study has several limitations needed to be addressed. First, although we incorporated two datasets from independent centers, the overall sample size remains relatively small. This limitation may have led to overestimation of model performance and raises concerns about generalizability to broader clinical settings. However, we showed that when SHAP is applied to an unseen dataset with comparable demographic characteristics, similar features capable of discriminating between MS and HC images are identified. Second, the lack of ground truth labels in the segmentation step may have led to less accurate outcomes [73]; nonetheless, we employed robust deep learning-based models that have previously demonstrated promising performance [43], along with appropriate adjustments in the pre- and post-processing stages. Third, we were not able to distinguish between MS eyes with and without a prior history of ON. This may have led to inaccurate estimations about the importance of each feature, since ON-positive eyes may probably harbor higher levels of neurodegenerative and vascular changes compared to ON-negative eyes, as observed in OCT [4] and OCT-A [15,16,17] studies. Such differences may have influenced vascular features like vessel width, density, and intensity—potentially exaggerating or masking group-level trends in the SHAP-based analysis. Stratification by ON history in future studies is necessary to more accurately isolate MS-specific vascular biomarkers. Fourth, the retinal changes occurring in the patient group may not exclusively be attributed to MS, but also to comorbid ocular or systemic disorders such as diabetes mellitus or hypertension. Although MS patients with such concurrent disorders were excluded in Ashtari et al.’s study [39], the source study of the Isfahan dataset, the same consideration is seemingly not employed by He et al. [40], the creators of the Johns Hopkins dataset. Finally, a more precise analysis of MS-specific features could have been achieved if retinal arteries and veins had been segmented separately. This would have allowed for the independent calculation of vessel parameters (e.g., width and intensity) for each vessel type, offering more detailed insight into vascular alterations in MS. Arteries and veins differ in physiological structure and function, and MS-related pathology may affect them differently. Therefore, including vessel-type-specific features in future work could improve interpretability and reveal alternative or complementary biomarkers. Of note, the artery–vein segmentation model proposed in the original study [43] did not yield satisfactory results on the external dataset, and its performance was further degraded in our study due to the incompatibility with additional pre- and post-processing steps (e.g., region growing and gap filling).

## 5. Conclusions

In this study, we identified key vessel morphological features within IR-SLO images that are clinically relevant for distinguishing between MS and healthy control individuals. The most important features were FD, VD in zone B, VD in zone C, VI and VW. This is in line with previous evidence suggesting that blood vessels can display pathologies within the retina among MS patients. These features were extracted following accurate segmentation by a deep learning model adapted from fundus to IR-SLO images. This successful adaptation enabled reliable retinal structure delineation and demonstrates that fundus-trained models can be effectively repurposed for IR-SLO, even with limited data—a valuable approach in data-constrained research settings. Therefore, such vessel characteristics obtained from IR-SLO images could provide useful biomarkers for diagnosing MS, should the results of this study be validated in future, multicenter studies on larger scales that consider distinguishing eyes with ON from those without, and separating retinal arteries from veins. Incorporating artery–vein classification and extracting vessel-type-specific features in future work could provide additional insight and help identify alternative or complementary biomarkers for MS diagnosis. In addition, future studies could explore more complex features such as deep-learned representations or vessel topology metrics (e.g., branching patterns or texture descriptors) to further enhance diagnostic accuracy and interpretability. This is of paramount importance since current diagnostic work-ups for MS include invasive, costly, and time-consuming procedures like magnetic resonance imaging and cerebrospinal fluid sampling.

## Figures and Tables

**Table 1:** Demographic characteristics of participants in Isfahan and Johns Hopkins datasets.

		Isfahan (*n* = 106)	Johns Hopkins (*n* = 35)	*p*-Value
Mean age (±SD)	MS	34.29 (±8.24)	41.97 (±8.77)	0.001 *
	HC	31.59 (±7.80)	35.77 (±13.03)	0.631
	All	32.48 (±8.01)	39.49 (±10.94)	0.001 *
Gender (female/male)	MS	34/1	17/4	0.040 *
	HC	55/16	12/2	0.490
	All	89/17	29/6	0.878

Abbreviations: MS, multiple sclerosis; HC, healthy control; SD, standard deviation. * shows statistical significance.

**Table 2:** Quantitative evaluation of segmentation performance for retinal vessels, optic disc, and optic cup using the adapted deep learning model on IR-SLO images.

Structure	Dice Coefficient (%)	F1 Score (%)	IoU (%)	Sensitivity (%)	Specificity (%)
Retinal Vessels	97.0	96.8	94.2	95.5	98.7
Optic Disc	91.0	90.6	84.5	89.2	97.3
Optic Cup	94.5	94.1	89.8	93.0	98.0

**Table 3:** Performance metrics of three simple machine learning models (SVM, RF, and XGBoost classifiers) for classification of MS using SLO images. Best results are bolded, revealing that the XGBoost classifier is the winning classifier.

Model	ACC	AUROC	AUPRC	F1	SP	SE	PR
SVM (kernel: RBF)	79.88 ± 5.73%	85.25 ± 6.55%	84.83 ± 8.64%	79.67 ± 5.97%	78.23 ± 9.98%	79.40 ± 12.88%	78.95 ± 6.94%
RF	78.99 ± 6.55%	85.09 ± 8.93%	85.16 ± 8.59%	78.80 ± 6.64%	76.52 ± 8.89%	79.91 ± 14.47%	77.58 ± 6.15%
**XGBoost**	**82.37 ± 4.91%**	**86.28 ± 8.12%**	**85.37 ± 8.32%**	**82.14 ± 5.06%**	**78.63 ± 9.56%**	**83.79 ± 11.12%**	**80.39 ± 6.55%**

## Data Availability

The Johns Hopkins dataset is available at: http://iacl.jhu.edu/Resources. The Isfahan dataset analyzed during the current study is not publicly available but can be obtained from the corresponding author on reasonable request, subject to ethical and institutional approval.

## Supplementary Materials

The following supporting information can be downloaded at: https://www.mdpi.com/article/10.3390/bioengineering12080847/s1, Figure S1: The IR-SLO image selected as the reference image for the histogram matching algorithm; Figure S2: Inversed matched IR-SLO images with a central reflection, shown as a dark strip on veins, and the image resulting from a morphological closing and opening operation; Figure S3: Visualization of an IR-SLO image, its optic disc candidate, and the result obtained from the blob algorithm; Figure S4: Visualization of the algorithm used for estimating the boundary of the optic disc candidate located on the right side of the IR-SLO image; Figure S5: A visualization of the IR-SLO image with varying background intensity variations, the reference image used for the histogram matching algorithm, and the IR-SLO image with a contrast level matched to that of the reference image; Figure S6: The region-growing algorithm used for vessel segmentation; Figure S7: Zone B and zone C in an IR-SLO image; Figure S8: The process of measuring FD for an IR- SLO image; Table S1: The feature sets extracted from the optic disc, optic cup and blood vessels in the IR-SLO images, along with their corresponding *p*-values; Table S2: The hyperparameters tuned by Optuna software for SVM, RF and XGBoost classifiers; Algorithm S1: The region-growing model proposed for addressing discontinuities in the binary vessel map. References [45,46,62] are cited in the supplementary materials.

## Abbreviations

The following abbreviations are used in this manuscript:

MS	Multiple Sclerosis
HC	Healthy Control
CNS	Central Nervous System
OCT	Optical Coherence Tomography
RNFL	Retinal Nerve Fiber Layer
GCIPL	Ganglion Cell-Inner Plexiform Layer
AD	Alzheimer’s Disease
PD	Parkinson’s Disease
IR-SLO	Infrared Scanning Laser Ophthalmoscopy
AI	Artificial Intelligence
CNN	Convolutional Neural Network
FI	Feature Importance
SHAP	SHapley Additive exPlanations
VD	Vessel Density
FD	Fractal Dimension
VI	Vessel Intensity
VW	Vessel Width
FS	Feature Selection
ML	Machine Learning
RF	Random Forest
SVM	Support Vector Machine
XGBoost	Extreme Gradient Boosting
ACC	Accuracy
AUROC	Area Under the Receiver Operating Characteristic
AUPRC	Area Under the Precision-Recall Curve
PR	Precision Recall
SE	Sensitivity
SP	Specificity
ROC	Receiver Operating Characteristic
RFE	Recursive Feature Elimination
EBM	Explainable Boosting Machine
t-SNE	t-distributed Stochastic Neighbor Embedding
