# Supplementary material for: Analyzing Retinal Vessel Morphology in MS Using Interpretable AI on Deep Learning-Segmented IR-SLO Images
Source: Bioengineering (Basel). 2025 Aug 6;12(8):847. doi: 10.3390/bioengineering12080847 (PMC12384046; doi:10.3390/bioengineering12080847)
Supplement: Supplementary file 1 [file bioengineering-12-00847-s001.zip › bioengineering-3755314-supplementary.pdf]

Article

Analyzing Retinal Vessel Morphology in MS Using Interpretable AI on Deep Learning-Segmented IR-SLO Images

Asieh Soltanipour [1,†], Roya Arian [2,†], Ali Aghababaei [1,3], Fereshteh Ashtari [4], Yukun Zhou [5,6,7], Pearse A. Keane [5] and Raheleh Kafieh [2,*]

[1] Medical Image and Signal Processing Research Center, Isfahan University of Medical Sciences, Isfahan, Iran
[2] Department of Engineering, Durham University, South Road, Durham, UK
[3] School of Medicine, Isfahan University of Medical Sciences, Isfahan, Iran
[4] Isfahan Neurosciences Research Center, Isfahan University of Medical Sciences, Isfahan, Iran
[5] NIHR Biomedical Research Centre for Ophthalmology, Moorfields Eye Hospital NHS Foundation Trust and UCL Institute of Ophthalmology, London, UK
[6] Centre for Medical Image Computing, University College London, London, UK
[7] Department of Medical Physics and Biomedical Engineering, University College London, London, UK
† These authors contributed equally.
* Correspondence: raheleh.kafieh@durham.ac.uk

Supplementary File Abstract

This Supplementary File is intended to complement the main article by providing additional figures or details that support or expand upon the findings and discussions presented therein. The content included herein offers readers a more comprehensive understanding of the research conducted and facilitates further exploration investigating whether there are any distinct features associated with Multiple Sclerosis (MS) detectable in infrared reflectance scanning laser ophthalmoscopy (IR-SLO) images, namely monochromatic fundus-like images frequently captured alongside OCT scans.

The sections are arranged in accordance with the order of the main manuscript for ease of reference. Each supplementary section mentioned in the main manuscript includes relevant additional figures, tables, or explanations, improving clarity and coherence.

The IR-SLO image chosen as a reference image

Figure S1 shows the IR-SLO image selected as a reference in the optic disc segmentation step. As can be seen, this is an IR-SLO image where the optic disc is visible in comparison to the background and the variations in background intensity are also negligible.

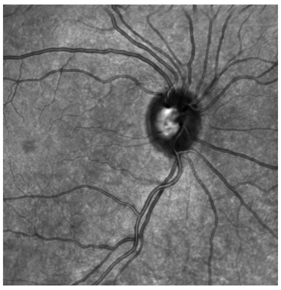

Figure S1. The IR-SLO image selected as the reference image for the histogram matching algorithm.

a. The impact of central reflection of blood vessels on the IR-SLO image

 https://doi.org/10.3390/bioengineering12080847

Figure S2 represents the central reflections of blood vessels on an IR-SLO image, along with the image obtained after removing their effects. As can be seen, the impact of this phenomenon appears as dark strips on veins.

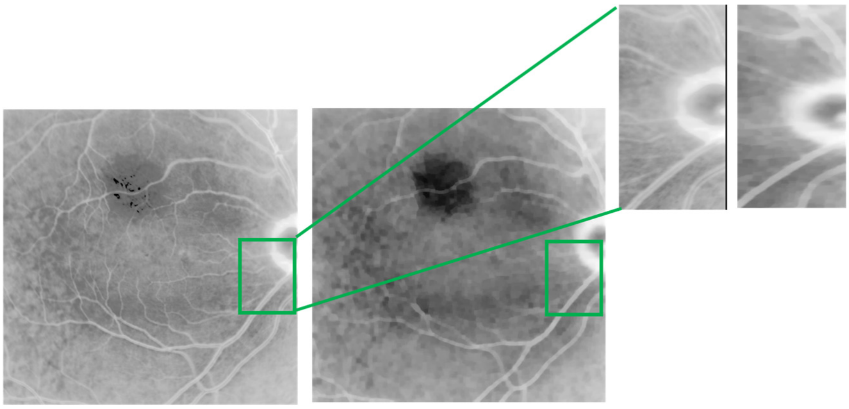

Figure S2. Inversed matched IR-SLO images with a central reflection, shown as a dark strip on veins (the left image), and the image resulting from a morphological closing and opening operation (the right image). The windows containing veins around the optic disc are also magnified.

b. The algorithm employed for calculating the boundary of the optic disc candidate located on the right side of the IR-SLO image

In acquiring the boundary of the optic disc candidate positioned on the right side of the image, the employed blob algorithm may occasionally encounter difficulties in accurate detection, as shown in the example illustrated in Figure S3.

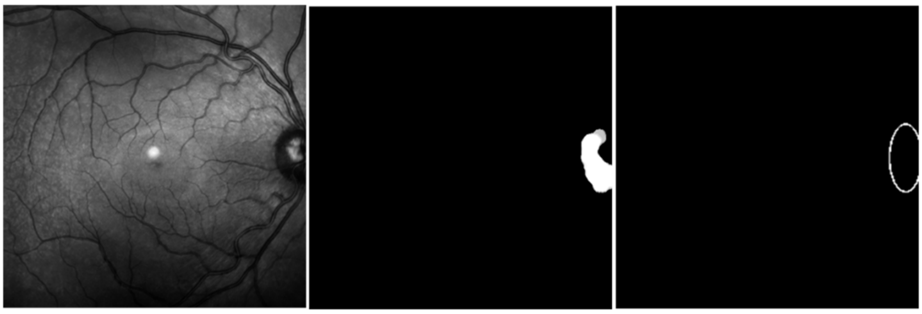

Figure S3. Visualization of an IR-SLO image (the left image), its optic disc candidate (the middle image), and the result obtained from the blob algorithm (the right image).

To tackle this challenge, we leveraged the information acquired from the blob algorithm to estimate the boundary of the optic disc candidate. Figure S4 illustrates the algorithm employed in cases where the blob algorithm alone struggled to measure the boundaries accurately. In the initial step, our task involved selecting three points—A, B, and C—on the boundary of the ellipse obtained from the blob algorithm, ensuring they were the maximum distance from each other. Subsequently, after computing the line segments AB and BC (coloured yellow and blue, respectively), we determined the perpendicular bisector for each of these segments, represented by two green lines. The intersection point of these calculated perpendiculars, denoted as D, was designated as the centre of the optic disc candidate. Finally, utilizing the centre (point D) and the length of the line segment BD, we measured the boundary of the optic disc candidate with the assistance of the blob algorithm. This information was then used to delineate the green-coloured circle on the IR-SLO image, representing the centre and radius of the optic disc candidate.

 https://doi.org/10.3390/bioengineering12080847

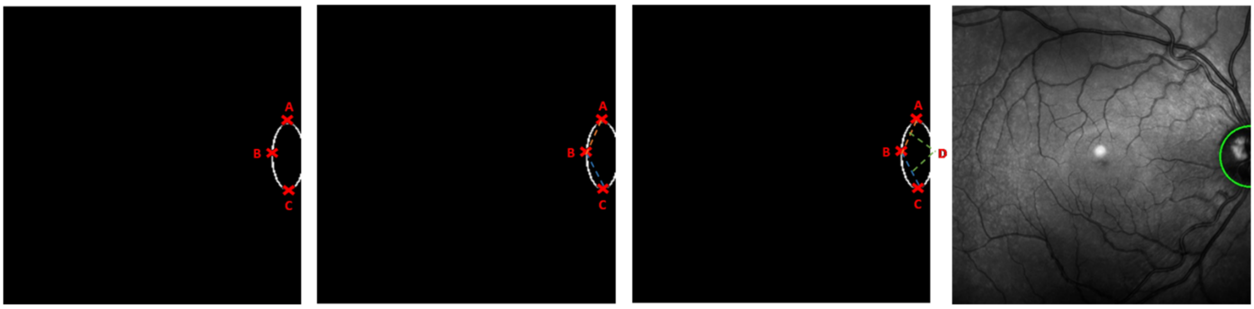

Figure S4. Visualization of the algorithm used for estimating the boundary of the optic disc candidate located on the right side of the IR-SLO image. The first to fourth columns, from left to right, represent the three points (A, B, and C) with the greatest distance from each other; the two line segments, AB and BC; their perpendiculars; and finally the boundary of the optic disc candidate, shown by the green coloured circle, respectively.

c. The reference image used in the vessel segmentation process

To segment blood vessels in the IR-SLO images within our dataset, we initially addressed variations in intensity values by manipulating the intensity histogram of the IR-SLO images. This manipulation aimed to mitigate the risk of false vessel pixel detection. To achieve this, we employed a histogram matching algorithm, utilizing the histogram intensity of a reference image to standardize the level of contrast across other IR-SLO images. Figure S5 A–C illustrate an IR-SLO image with intensity variations, a reference image, and the resulting modified contrast level of the IR-SLO image when aligned with the reference image, respectively. The selection of the reference image took into account not only the high-intensity values distinguishing the vascular tree from the background but also the minimal-intensity variation among background pixels.

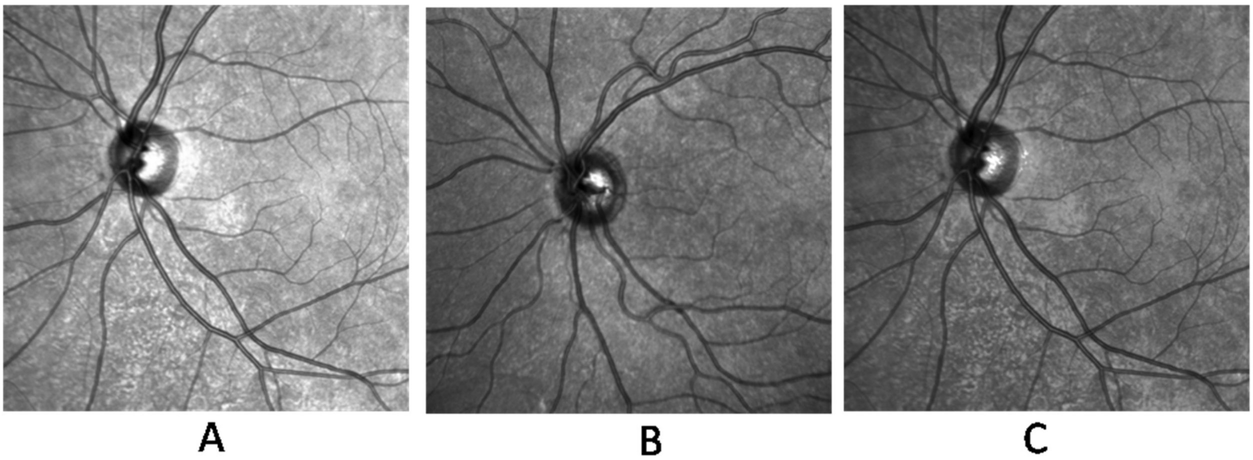

Figure S5. Part A: a visualization of the IR-SLO image with varying background intensity variations. Part B: the reference image used for the histogram matching algorithm. Part C: the IR-SLO image with a contrast level matched to that of the reference image.

d. A brief description of the region-growing algorithm

Figure S6 represents the region-growing algorithm. This algorithm includes some seed pixels in an image and progressively expands regions from these pixels by iteratively incorporating unassigned neighbouring pixels that meet specific criteria and align with the existing regions of the seed pixels. To accomplish this, we initially computed a skeleton image of the blood vessels segmented from the previous step. Subsequently, we

 https://doi.org/10.3390/bioengineering12080847

constructed an undirected vessel graph and identified terminal or end nodes as pixels belonging to the vessel skeleton with only one neighbouring skeleton pixel (1).

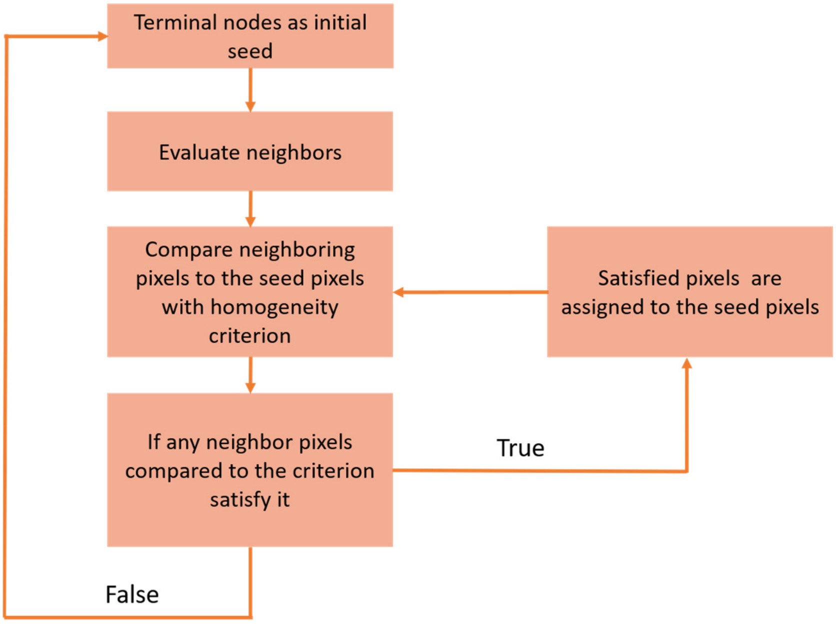

Figure S6. The region-growing algorithm used for vessel segmentation.

Algorithm S1. The region-growing model proposed for addressing discontinuities in the binary vessel map.

Algorithm 1 Region-growing

---

for each terminal point in skeleton of blood vessels:

threshold = intensity of node p_i

seed = p_i

S1=∅

new_seed = ∅

If intensity of the seed > 40 :

ALGORITHM A

neighboring set=neighboring pixels of seed not belonging to vessel

If there exists a pixel in the neighboring set whose intensity difference with the threshold < 10:

new_seed.append (neighboring pixel with the lowest difference value)

S1.append (the other neighbors satisfying the criterion)

threshold = mean (intensity of the seed, intensity of pixels in the new_seed)

Seed=neighboring pixels with the lowest difference

repeat algorithm A until no neighboring pixels satisfy the criterion.

ALGORITHM B

S = S1

---

 https://doi.org/10.3390/bioengineering12080847

S1 = Ø

for each point in S:

threshold = the intensity of $P_{S1,i}$

new_seed = Ø

If the intensity of $P_{S1,i}$ > 40 :

Do algorithm A

repeat algorithm A until no neighboring pixel satisfies the criterion

repeat algorithm B until S1=Ø

e. Zone B and zone C from the centre of the optic disc

In the current work, two standard areas centred on the optic disc of the IR-SLO images were used for vascular feature measurements, namely Zone B and Zone C. Figure S7 illustrates these two areas on a retinal image, where Zone B defines a circumferential region 0.5 to 1 optic disc diameter from the optic disc margin, and Zone C is associated with a circumferential region 0.5 to 2 optic disc diameters from the disc margin.

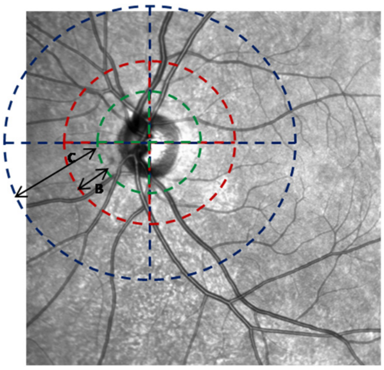

Figure S7. Zone B and zone C in an IR-SLO image. The green circle shows the optic disc margin; line b, the line between the green and red circles, and line c, the line between the green and blue circles, indicate zone B and zone C, respectively.

f. Features used for the classification of IR-SLO images into two classes, namely MS and HC

Table S1. The feature sets extracted from the optic disc, optic cup and blood vessels in the IR-SLO images, along with their corresponding *p*-values. Features with *p*-values < 0.05 are highlighted.

**Manual features extracted from IR-SLO images**		**Definition**	***p*-Values**
Features From Optic Disc/Cup	Disc Width	The width of the extracted optic disc in the IR-SLO images.	1.448e-01
	Cup Width	The width of the extracted cup in the IR-SLO images.	**2.156e-06**
	Disc Width-to-Cup Width	The ratio of disc width to cup width	**3.181e-06**
Vascular Features From Whole Image	Average Width	The ratio of blood vessels to their skeleton in the IR-SLO images.	**7.405e-08**
	Fractal Dimension	The metric of the self-similarity and complexity of blood vessels following a change in the length of scales.	**1.309e-09**
	Vessel Density	The ratio of the blood vessel to the total area in an IR-SLO image.	**7.097e-04**

 https://doi.org/10.3390/bioengineering12080847

	Vessel Intensity	The proportion between the intensity of vessels and the intensity of the whole image.	**4.204e-04**
	Distance Measure Tortuosity	The ratio of the actual path length to the linear distance between the endpoints of a skeletal vessel segment.	2.090e-01
	Squared Curvature Tortuosity	A metric defined as the sum of all integrals measured from the square of the curvature along the path of the vessel segment.	1.026e-01
	Tortuosity Density	A metric defined by multiplying the number of inflexion points with the sum of the distance measurements between the change points in a piece of vessel skeleton.	2.706e-01
VascularF From Zone B and Zone C	Average Width	The ratio of blood vessels to their skeleton in the IR-SLO images in zones B and C.	Zone B : 4.047e-01<br>Zone C: 3.898e-01
	Fractal Dimension	The metric of the self-similarity and complexity of blood vessels under a change in length scales in zones B and C.	Zone B: 6.177e-02<br>Zone C: 5.215e-02
	Vessel density	The ratio of the blood vessel to the total area in an IR-SLO image in zones B and C.	**Zone B: 6.258e-04**<br>**Zone C: 2.184e-03**
	Linear Regression Tortuosity	A metric measuring the factor (between 0 and 1) when each segment of the vessel skeleton has a linear pattern.	Zone B: 4.722e-01<br>Zone C: 3.897e-01
	Distance Measure Tortuosity	The ratio of the actual path length to the linear distance between the endpoints of a skeletal vessel segment.	Zone B: 8.141e-01<br>Zone C: 5.955e-01
	Squared Curvature Tortuosity	A metric defined as the sum of all integrals measured from the square of the curvature along the path of the vessel segment.	Zone B: 7.268e-01<br>Zone C: 6.526e-01
	Tortuosity Density	A metric defined as multiplying the number of inflexion points with the sum of the distance measurements determined between the change points in a piece of vessel skeleton.	Zone B: 4.009e-01<br>Zone C: 4.318e-01

g. A brief description of the algorithm for fractal dimension measurements

Fractal dimension (FD) is a score measuring how the details of an object change at different magnifications. Thus, FD shows self-similarity and complexity under a change in length scales (2). This measurement was considered a potential biomarker to recognize some diseases, like diabetes and hypertension, through a description of branching vascular distribution in two-dimensional space (3). There are different methods to calculate FD, including box counting, the mass–radius relation, the two density–density points, or the pair correlation function method (2). Box-counting, the most common way to measure FD, was the method used in this study. It works by overlaying the binary image (the map of segmented vessels) with a grid of boxes of side length ε and counting the number of boxes containing a part of the vessel tree. This process is repeated under different values of ε to obtain more and more fine details of the vascular tree from the covering. Finally, the box-counting dimension can be calculated through the following formulate, in which N(∈) represents the number of boxes containing a vascular tree (3):

$$D_{box} = \lim_{\in \to 0} \frac{\log N(\in)}{\log(1/_{\in})} \quad (1)$$

Figure S8 shows the process of calculating FD for an IR-SLO image based on the box-counting method. The IR-SLO image and its segmented blood vessel map are shown in

the first and second columns, respectively. The third and fourth columns represent what is stated in Equation 1. It should be noted that min–max normalization is applied to normalize the FD value, transforming its range into [0,1) without altering the significance of this feature.

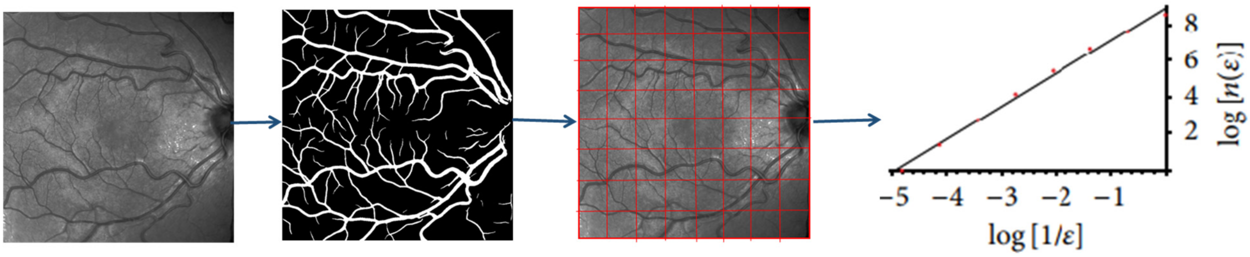

Figure S8. The process of measuring FD for an IR- SLO image.

h. The hyperparameters optimized using Optuna

Table S2. The hyperparameters tuned by Optuna software for SVM, RF and XGBoost classifiers.

Model	Optimal hyper parameters
SVM (kernel : RBF)	C = 7072.776159189032<br>Gamma = 3.9196303352470423
RF	max_depth = 10<br>n_estimators = 147<br>min_samples_split = 10<br>min_samples_leaf = 2<br>criterian = gini
XGBoost	objective = binary :logistic<br>tree method = auto<br>booster = gbtree<br>lambda = 0. 6262819848273675<br>alpha = 0. 002417237009326353<br>subsample = 0. 6816657652094626<br>colsample_bytree = 0. 3288592429227775<br>max_depth = 3<br>min_child_weight = 4<br>eta = 0. 02168530523973617<br>gamma = 0. 01720205835153038<br>grow_policy = deptwise

References

1. Lyu, X.; Yang, Q.; Xia, S.; Zhang, S. *Construction of Retinal Vascular Trees via Curvature Orientation Prior*; IEEE: Piscataway, NJ, USA, 2016; pp. 375–382.
2. Falconer, K. *Fractal Geometry: Mathematical Foundations and Applications*, 3rd ed; Wiley: Hoboken, NJ, USA, 2013; p. 386.
3. Zekavat, S.M.; Raghu, V.K.; Trinder, M.; Ye, Y.; Koyama, S.; Honigberg, M.C.; Yu, Z.; Pampana, A.; Urbut, S.; Haidermota, S.; et al. Deep Learning of the Retina Enables Phenome- and Genome-Wide Analyses of the Microvasculature. *Circulation* **2022**, *145*, 134–150.

Disclaimer/Publisher's Note: The statements, opinions and data contained in all publications are solely those of the individual author(s) and contributor(s) and not of MDPI and/or the editor(s). MDPI and/or the editor(s) disclaim responsibility for any injury to people or property resulting from any ideas, methods, instructions or products referred to in the content.

 https://doi.org/10.3390/bioengineering12080847
